# Circulating Angiogenic Cell Dysfunction in Patients with Hereditary Hemorrhagic Telangiectasia

**DOI:** 10.1371/journal.pone.0089927

**Published:** 2014-02-25

**Authors:** Liana Zucco, Qiuwang Zhang, Michael A. Kuliszewski, Ivana Kandic, Marie E. Faughnan, Duncan J. Stewart, Michael J. Kutryk

**Affiliations:** 1 Division of Cardiology, Keenan Research Center for Biomedical Science at the Li Ka Shing Knowledge Institute, St. Michael’s Hospital, University of Toronto, Toronto, Ontario, Canada; 2 Division of Respirology, Keenan Research Center for Biomedical Science at the Li Ka Shing Knowledge Institute, St. Michael’s Hospital, University of Toronto, Toronto, Ontario, Canada; 3 Ottawa Health Research Institute, Ottawa, Ontario, Canada; 4 St. George’s Hospital Trust, South Thames Foundation School, London, United Kingdom; European Institute of Oncology, Italy

## Abstract

Hereditary hemorrhagic telangiectasia (HHT) is an autosomal dominant vascular disorder. Circulating angiogenic cells (CACs) play an important role in vascular repair and regeneration. This study was designed to examine the function of CACs derived from patients with HHT. Peripheral blood mononuclear cells (PBMNCs) isolated from patients with HHT and age- and gender-matched healthy volunteers were assessed for expression of CD34, CD133 and VEGF receptor 2 by flow cytometry. PBMNCs were cultured to procure early outgrowth CACs. Development of endothelial cell (EC) phenotype in CACs was analyzed by fluorescence microscopy. CAC apoptosis was assayed with Annexin V staining, and CAC migration assessed by a modified Boyden chamber assay. mRNA expression of endoglin (*ENG)*, activin receptor-like kinase-1 (*ACVLR1 or ALK1*) and endothelial nitric oxide synthase (*eNOS*) in CACs was measured by real time RT-PCR. The percentage of CD34+ cells in PBMNCs from HHT patients was significantly higher than in PBMNCs of healthy controls. CACs derived from patients with HHT not only showed a significant reduction in EC-selective surface markers following 7-day culture, but also a significant increase in the rate of basal apoptosis and blunted migration in response to vascular endothelial growth factor and stromal cell-derived factor-1. CACs from HHT patients expressed significantly lower levels of *ENG*, *ALK1* and *eNOS* mRNAs. In conclusion, CACs from patients with HHT exhibited various functional impairments, suggesting a reduced regenerative capacity of CACs to repair the vascular lesions seen in HHT patients.

## Introduction

Hereditary hemorrhagic telangiectasia (HHT), an autosomal dominant vascular dysplasia, affects approximately 1 in 5–8000 people [1], [2]. Patients with HHT often show characteristic mucocutaneous telangiectasia, and common clinical presentations are recurrent epistaxis, gastrointestinal bleeding, visceral arteriovenous malformations and iron deficiency anemia [3]. The most common forms of HHT, HHT1 and HHT2, have been linked to mutations in the endoglin (*ENG*) and activin receptor-like kinase 1 (*ACVLR1* or *ALK1*) genes respectively, both encoding putative receptors for the transforming growth factor-beta (TGF-β) superfamily that play a critical role for the proper development of the blood vessels [4]–[6]. Mutations in *SMAD4* have also been identified in a subset of patients with a combined syndrome of HHT and juvenile polyposis [7].

The TGF-β signal transduction pathways involve type I and type II serine/threonine kinasereceptors. TGF- β ligands bind type II receptors, leading to the subsequent recruitment of type I receptors to the ligand/receptor II complex. Phosphorylation of the type I receptors, results in phosphorylation/activation of intracellular effectors known as Smads [8], [9]. ALK1 is a type I receptor while endoglin, primarily expressed in endothelial cells (ECs), is an accessory receptor which collaborates with ALK1 to promote cell migration and proliferation [10]–[13]. It has been well recognized that aberrant TGF-β signaling caused by *ENG* or *ALK1* mutations affects primarily ECs in HHT patients [14]. Protein levels of ENG and ALK1 have been demonstrated to be decreased in ECs from patients with HHT [15], [16], which results in altered TGF-β signaling, thought to be responsible for the endothelial dysfunction which contributes to the vascular lesions typical for HHT. Circulating angiogenic cells (CACs), sometimes referred to as endothelial progenitor cells (EPCs), are derived from bone marrow and comprise a fraction of the circulating mononuclear cell population [17]–[19]. They can differentiate into mature, functional endothelial-like cells that can be incorporated into vessels and can produce angiogenic factors that contribute to vascular repair and regeneration. Most studies focussed on the exploration of EPCs for therapeutic angiogenesis or the examination of EPCs as potential biomarkers for cardiovascular disease, have used cells that are generated by the culture of peripheral blood mononuclear cells on fibronectin in vascular endothelial growth factor (VEGF)-containing medium [20]–[22]. These EPCs are not homogeneous but rather constitute a heterogenic population that mainly originates from myeloid hematopoietic cells [21]–[26]. Furthermore, phenotypic analysis has revealed few EPCs expressing stem/progenitor-cell markers [26]. These findings make it controversial to name these cells “EPCs” and the term “CACs” has been suggested, given that these cells have been shown to promote angiogenesis and vascular repair in various experimental settings [27]–[29]. We hypothesized that CAC function from patients with HHT is impaired, and can be demonstrated in a series of in vitro cell function assays.

## Materials and Methods

### Ethics Statement

All protocols involving human samples were approved by the Research Ethics Board of St. Michael’s Hospital, University of Toronto, in accordance with The Code of Ethics of the World Medical Association (Declaration of Helsinki).

### Patient Recruitment

Informed written consent for study participation was obtained from all patients and healthy volunteers. We enrolled a total of 35 patients clinically diagnosed with HHT with 31 with known mutations in either *ENG* or *ALK1*. Healthy age- and gender-matched volunteers (n = 33) served as controls.

### Cell Isolation and Culture

Peripheral blood mononuclear cells (PBMNCs) were isolated from 80 ml of venous blood by Ficoll density gradient centrifugation. After 2 washes with phosphate buffered saline (PBS), PBMNCs were directly analyzed by flow cytometry for the expression of CD34, CD133 and VEGFR2, or plated at a density of 0.75×10^6^/cm^2^ in human fibronectin-coated dishes or slides and cultured in endothelial cell basal medium supplemented with 20% human serum and various growth factors (EGM-2MV, Cambrex) to procure early outgrowth CACs. After 3 days non-adherent cells were removed and fresh culture medium was supplied. Cells were analyzed at day 7 with change of medium every other day.

### Flow Cytometry

Freshly isolated PBMNCs were analyzed for expression of CD34, CD133 and vascular endothelial growth factor receptor 2 (VEGFR2) by flow cytometry. A FITC-anti-human CD34 antibody (Diatec, 1∶100 dilution), a PE-anti-human VEGFR2 antibody (R&D Systems, 1∶50 dilution) and a PE-anti-human CD133 antibody (Miltenyi Biotec, 1∶100 dilution) were used. PBMNCs were incubated with an appropriate antibody alone or in combination, along with the appropriate isotype controls, at 4°C for 30 minutes in the dark. Following 2 subsequent washes with PBS, 1×10^6^ cells were resuspended in 500 µl of PBS and analyzed using a Beckman Coulter flow cytometer.

### Immunofluorescence Microscopy

Following a 7-day culture, development of an EC phenotype in CACs was assessed by cell uptake of Dil-Ac-LDL, binding of UEA-Lectin, and detection of *VEGFR2* expression, as previously described [30]–[32]. For the Dil-Ac-LDL up-take assay, CACs were washed once with PBS and fluorescent dye labeled Dil-Ac-LDL (10 µg/ml), dissolved in serum free medium, was added to cells. Cells were cultured for 4 hours followed by 2 washes with PBS and fixed with 2% paraformaldehyde in PBS for 10 minutes. After 2 washes with PBS, FITC-UEA-1 (1∶200 dilution, Sigma) was added to cells and incubated overnight at 4°C. Following this incubation, cells were washed, counter stained with the nuclear marker ToPro3 (Molecular Probes) and mounted with VectaShield Mounting Medium. VEGFR2 immunostaining was done as follows; cells were washed 2 times with PBS and fixed with 2% paraformaldehyde in PBS containing 0.5% Triton X-100. After 2 washes with PBS, anti-VEGFR2 antibody (Chemicon, 1∶200 dilution) was added to cells and incubated at room temperature for 1 hour. Cells were washed with PBS followed by incubation with a FITC-conjugated secondary antibody (Invitrogen, 1∶1000 dilution). After 2 washes with PBS, cells were mounted using VectaShield with propidium iodide (PI). Dil-Ac-LDL/FITC-UEA-1 double positive cells and VEGFR2 positive cells were observed by confocal microscopy (Leica Microsystems and BioRad Laboratories), and the percentage of positive cells was calculated from at least 5 different fields against total cell number. The results were compared between CACs from HHT patients and healthy controls.

### Cell Apoptosis Assay

Basal CAC apoptosis after a 7-day culture, and CAC apoptosis induced by TNF-α or serum starvation were analyzed using an AnnexinV FLOUS staining kit (Roche Applied Science, Indianapolis, IN, USA) and flow ctyometry. CACs were exposed to TNF α (20 ng/ml) or serum free medium (SF) for 24 hours. Adherent CACs were detached from culture slides using PBS/1 mM EDTA, transferred to 1.5 ml eppendorf tubes and gently centrifuged at 300 g for 10 minutes. CACs were then re-suspended in 400 µl of Binding Buffer alone or in 400 µl of Binding Buffer mixed with 5 µl of FITC-conjugated Annexin V and 5 µl of 7-Amino-Actinomycin. After incubation in the dark for 15 minutes, cells positive for FITC-Annexin V were analysed by a Beckman Coulter flow cytometer.

### CAC Migration Assay

Cell migration was assessed using a modified-Boyden chamber assay. CACs were detached from culture slides, washed with PBS and resuspended at a density of 5×10^5^/ml in migration medium (EBM-2 medium containing 0.5% BSA). 500 µl (2.5×10^5^) of cells were then added into the top chamber of the modified Boyden chamber apparatus (BD Biosciences, 8 µm pores). The chemoattractants VEGF165 (Sigma) or stromal cell derived factor 1 (SDF1, R&D Systems), used to promote migration, were prepared with the migration medium at concentrations of 50 ng/ml and 100 ng/ml, respectively. 500 µl of chemoattractant or migration medium alone was added to the lower chamber. Following a 5 hour incubation period at 37°C migratory, cells present on the underside of the insert were fixed and stained using Diff Quik (Fisher Scientific) and visualized by light microscopy. Images were acquired for 5 randomly selected fields and the mean number of cells from these fields was determined. Data are presented as fold change in cell migration towards chemoattractant compared to the respective control basal migration.

### RNA Isolation, Reverse Transcription (RT) and Real Time PCR

Quantitative RT-PCR was performed to assess *ENG*, *ALK1* and *eNOS* mRNA expression in CACs. Total RNA was isolated using the RNAeasy kit (Qiagen) according to the manufacturer’s instructions. Briefly, 0.5–1×10^6^ cells were lysed in 700 µl Qiazol lysis reagent followed by extraction with 140 µl of chloroform. The aqueous phase containing RNA was transferred to a new microtube and 1.5 volumes of 100% ethanol was added and mixed. The sample was loaded into an RNeasy mini spin column followed by brief centrifugation. The column was washed with RWT and RPE buffers, and 40 µl of nuclease-free water was used to elute the RNA. The RNA sample was quantified by spectrophotometry. The first strand cDNA was generated by reverse transcription (RT) reaction using an Omniscript RT Kit (Qiagen). The RT components in a total volume of 20 µl contained 2 µl of 5 mMdNTPs, 5 µl of random primer (300 ng/ml), 2 µl of 10× reaction buffer, 1 µl of Reverse Transcriptase (4 units), 1 µl of RNase inhibitor (1 unit), 1 µg of RNA and nuclease-free ddH_2_O. RT was done by incubation at 37°C for 1 hour followed by enzyme inactivation at 65°C for 15 minutes. Real-time PCR was performed using the SYBR green kit (Applied Biosystems Inc.) on a 7900 HT Sequence Detection System (Applied Biosystems Inc.) with beta-actin gene used as an internal control. The reaction, in a 20 µl volume, contained 10 µl of 10x reaction buffer, 2 µl of RT product, 0.2 µM of gene specific primers and ddH_2_O. PCR was accomplished in three stages: stage 1; 50°C for 2 minutes, stage 2; 95°C for 10 minutes, stage 3; 95°C for 15 seconds followed by 60°C for 1 minute. Stage 3 was repeated for 40 cycles. A dissociation curve was set up to determine the specificity of each gene amplification. The relative level of each mRNA normalized to beta actin was determined using the 2^−ΔΔCT^ method. Primers for each individual gene were designed to span introns to eliminate detection of genomic contamination. The following primer sequences were used:

*ENG*; Forward 5′-AGCTGACTCTCCAGGCATCC-3′,

Reverse 5′-GCAGCTCTGTGGTGTTGACC-3′,

*ALK1*; Forward 5′-GTGAGAGTGTGGCCGTCAAG-3′,

Reverse 5′-CATGTCTGAGGCGATGAAGC-3′,

*eNOS*; Forward 5′-ACC CTC ACC GCT ACA ACA TC-3′,

Reverse 5′-GCC TTC TGC TCA TTC TCC AG-3′,

*β-actin*; Forward 5′-AGCCTCGCCTTTGCCGA-3′,

Reverse 5′-CTGGTGCCTGGGGCG-3′.

### Statistical Analysis

As a normal Gaussian distribution could not be assumed, significance of differences was determined using the non-parametric Mann-Whitney test. A student’s paired T-test was performed when analyzing results from paired migration assays. Data is presented as median ± interquartile range with *P*<0.05 indicating a statistically significant result.

## Results

Freshly isolated PBMNCs from HHT patients and healthy controls were immediately analyzed by flow cytometry for the expression of CD34, CD133 and VEGFR2, or were cultured as described above to procure early outgrowth CACs that were further studied and compared between patients and healthy controls. The results for HHT1 and HHT2 patient samples were combined as no difference between these two sub-groups were identified.

### Study Subjects

A total of 35 patients clinically diagnosed with HHT (31 with known mutations in either *ENG* or *ALK1*) and 33 healthy volunteers were recruited. Patient demographics were summarized in Table 1.

**Table 1:** Summary of Patient Demographics.

	Males	Females	AverageAge (M)	AverageAge (F)	Mutation
Controls	11	22	34.45±6.52	40.09±8.53	None
HHT	9	26	48.90±21.02	43.60±14.24	*ENG* (n = 22)*ALK-1* (n = 9)*SMAD4* (n = 2)Unknown (n = 2)

### Analysis of Circulating PBMNCs

**Figure 1:**
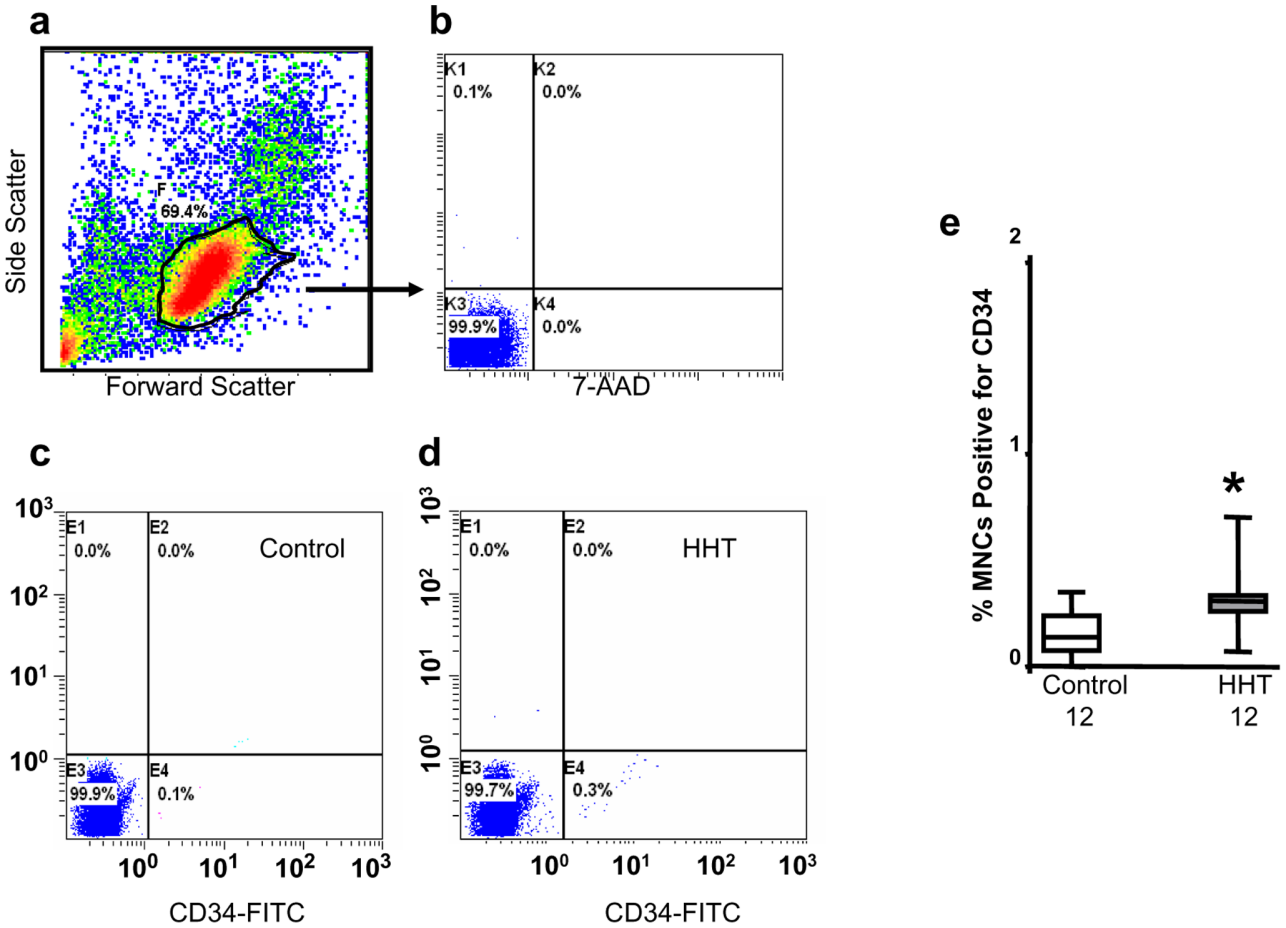
Increased CD34+ cells in PBMNCs isolated from HHT patients. Flow cytometric analysis showed that PBMNCs consisted of cells with different size and granularity (a), monocytic/lymphocytic population stained by fluorescent dye 7-AAD indicated over 99% cell viability (b). Manually gated cells for CD34 staining analysis were performed. A representative dot plot of CD34 analysis for a healthy volunteers and a HHT patient was shown in c and d, respectively. About 0.3% CD34+ cells were identified in PBMNCs from HHT patients, which was significantly higher than that in the control samples (e). *p<0.05, compared with healthy controls.

Flow cytometry was performed directly after PBMNC isolation. The forward scatter/side scatter density plot demonstrated variation in size and granularity of the cell types present within the PBMNC population (Figure 1a). A manual gate was drawn around the monocytic/lymphocytic population and maintained constant during each run. Absence of 7-AAD staining demonstrated 99% viability of this population (Figures 1a and 1b). Results from subsequent gated flow cytometric analyses revealed an increase in circulating CD34^+^ cells in patients with HHT when compared to controls (Figures 1c and 1d). The percentages of CD34^+^ positivecellswere 0.30±0.04% (n = 12) and 0.16±0.04% (n = 12) for HHT patients and controls respectively (*P*<0.05, Figure 1e). There were no significant differences observed for CD133+ cells or double CD34+/VEGFR2+ cells between patients and healthy subjects (data not shown).

### Phenotypic Study of Early Outgrowth CACs

**Figure 2:**
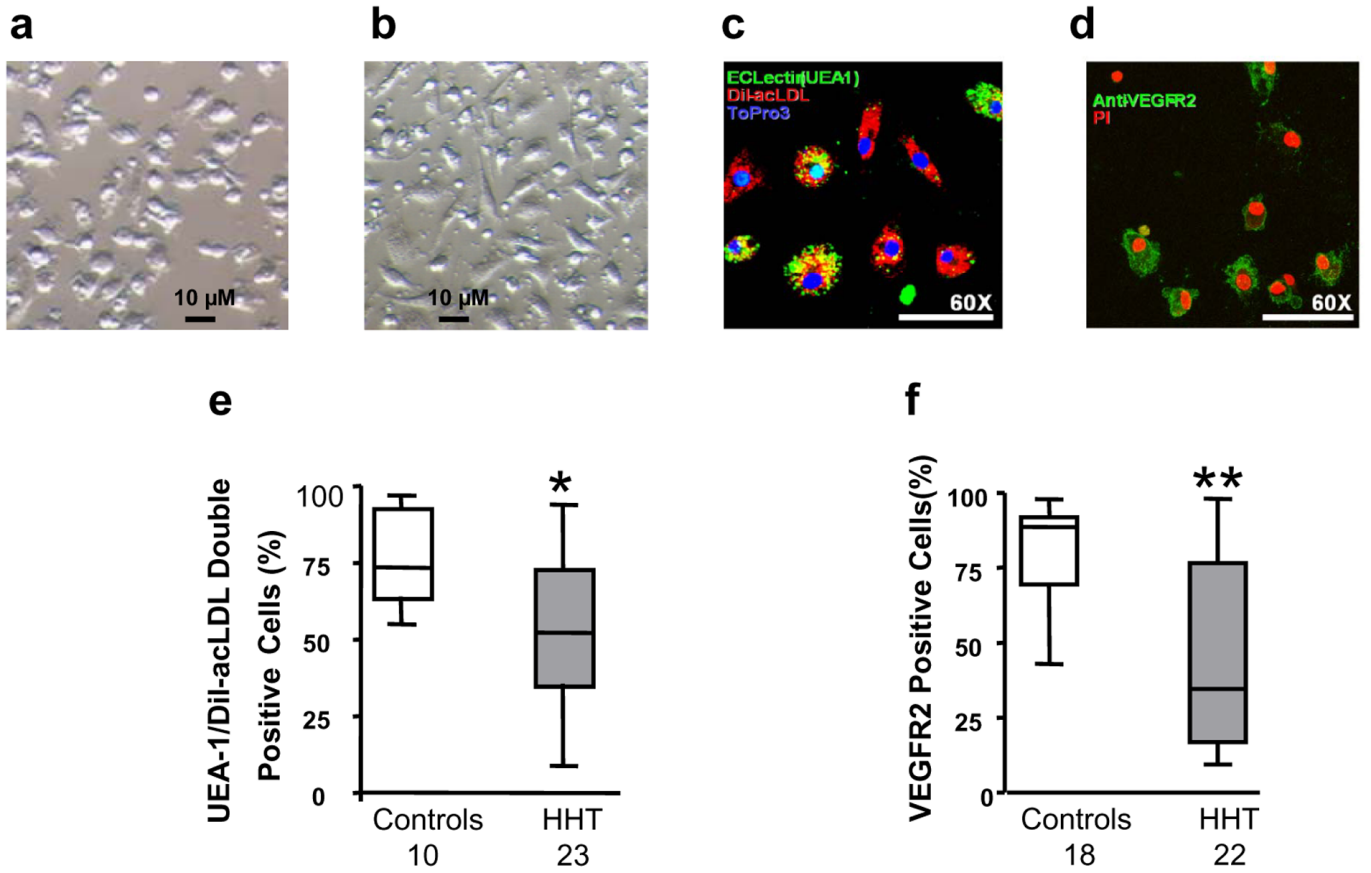
Reduced development of endothelial cellular phenotypes in CACs from HHT patients. CACs derived from patients with HHT appeared smaller in size and more rounded (a) when compared to the spindle-shaped, elongated morphology of CACs derived from control subjects (b). Dil-Ac-LDL/UEA-1 double positive cells (a representative image shown in c. Orange: double positive; green: UEA-1 positive; red: Dil-Ac-LDL positive; blue is nuclear stain) and VEGFR2 positive cells (a representative image shown in d. Green: VEGFR2 positive; red is nuclear stain) in CACs were analyzed by immunofluorescence microscopy. The results showed that percentage of Dil-Ac-LDL/UEA-1 double positive cells and percentage of VEGFR2 positive cells, both were significantly lower in CACs isolated from HHT patients (e and f). *p<0.05, compared with healthy controls. **p<0.01, compared with healthy controls.

Semi-confluent CAC cultures were seen in the 2 groups after 7 days. These CACs expressed minimal CD11b (<1%), a macrophage marker, as determined by flow cytometric analysis (data not shown). CACs derived from patients with HHT appeared smaller in size and more rounded (Figure 2a) when compared to the spindle-shaped, elongated morphology of CACs derived from control subjects (Figure 2b). Development of an endothelial cell phenotype in CACs was assessed by cell uptake of Dil-Ac-LDL, FITC-UEA-1 staining and expression of VEGFR2 with immunofluorescence microscopy. For quantification, a minimum of 5 images were taken at random, and the percentages of Dil-Ac-LDL/UEA-1 double positive cells (Orange, Figure 2c) and VEGFR2 positive cells (Green, Figure 2d) were calculated against the total cell number. The results showed that Dil-Ac-LDL/UEA-1 double positive cells were 53.4±5.1% (n = 23) and 75.0±3.9% (n = 15) for HHT patients and healthy controls respectively (*p*<0.05, Figure 2e), and that VEGFR2 positive cells were 46.0±6.5% (n = 22) and 76.9±3.8% (n = 21) for HHT patients and healthy subjects respectively (*p*<0.01, Figure 2f).

### CAC Apoptosis

**Figure 3:**
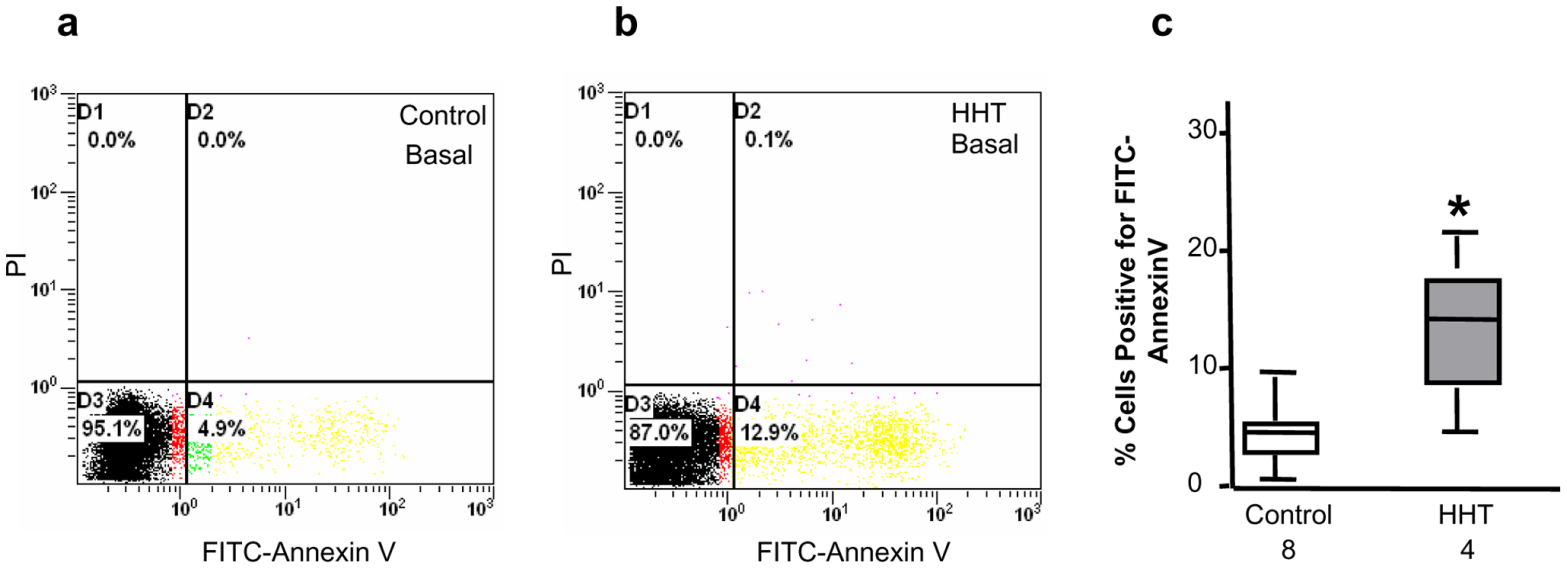
Elevated basal cell apoptosis levels in CACs from HHT patients. Basal cell apoptosis in CACs after a 7-day culture was analyzed by Annexin V staining and flow cytometry. A representative dot plot of flow cytometric analysis for healthy and HHT CAC apoptosis was shown in panels a and b, respectively. Statistic analysis demonstrated that the levels of basal cell apoptosis in CACs from HHT patients were significantly higher than those in CACs of healthy individuals (c). *p<0.05, compared with healthy controls.

CAC basal apoptosis was measured by AnnexinV staining and flow cytometry. Figures 3a and 3b show representative dot plots of flow cytometric apoptosis analysis for CACs from a healthy control and a HHT patient, respectively. CACs from patients with HHT had an apoptotic rate of 13.65±3.52% (n = 4), which was significantly higher than that measured for healthy CACs (4.5±0.96%; n = 8, *p*<0.05; Figure 3c). Futhermore, cell apoptosis measured after exposure of CACs to TNFα (20 ng/ml) or serum free medium for 24 hours showed significant increases of cell apoptosis in both patients’ and healthy controls’ CACs. However, there was no difference between HHT and control CACs in their apoptotic response to TNFα or serum starvation stress (data not shown).

### CAC Migration

**Figure 4:**
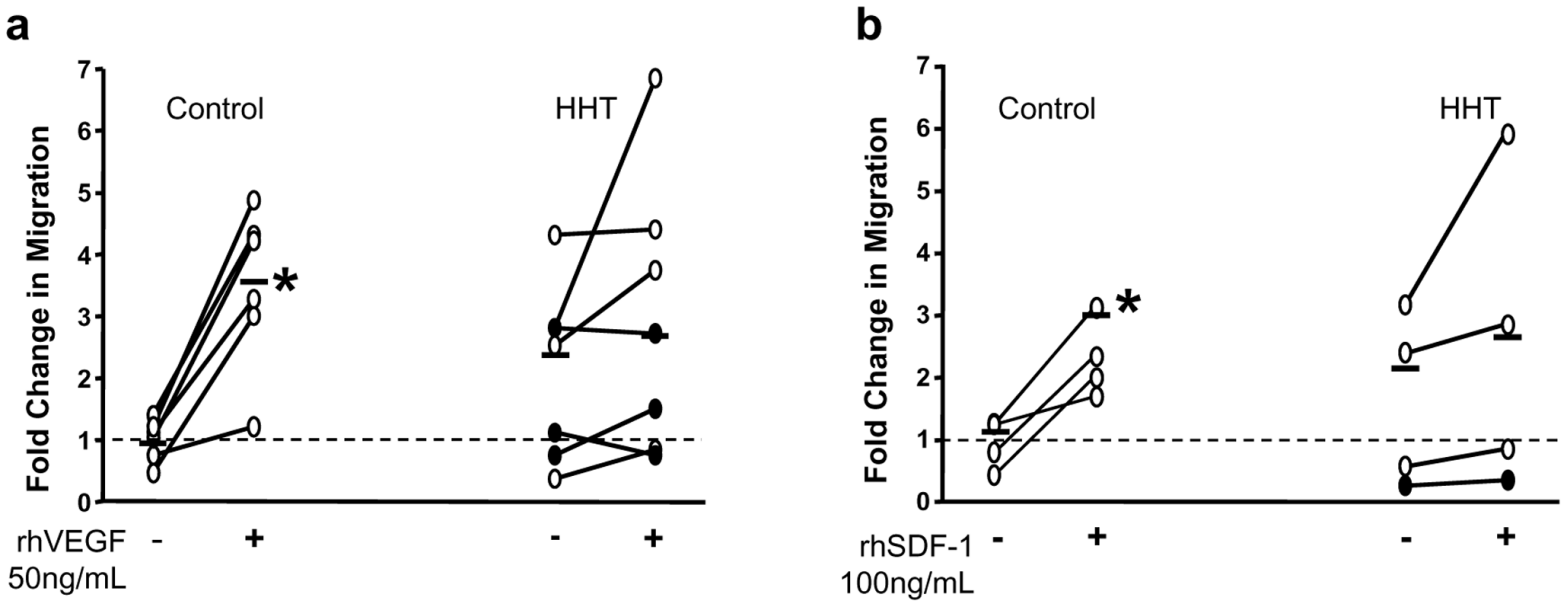
Suppressed cell migration towards VEGF and SDF1 in CACs from patients with HHT. Cell migration towards VEGF and SDF1 in healthy CACs increased by 4-folds and 3-folds, respectively, over the non-treated cells (a and b). In contrast, both VEGF and SDF1 induced HHT CAC migration was barely increased over the non-treated cells (a and b). *p<0.05, compared with cells without VEGF or SDF1 treatment.

CAC migration towards VEGF and SDF1 was assessed using a modified Boyden chamber assay. As shown in Figure 4a, in the presence of VEGF, a 4-fold increase of cell migration was observed for CACs from healthy controls (n = 6, *p<*0.05). No significant increase in cell migration towards VEGF was seen for CACs from HHT patients. Similarly, SDF1 induced a 3-fold increase of cell migration for CACs from healthy volunteers (Figure 4b). In contrast, there was no significant increase in cell migration towards SDF1 measured in CACs from HHT patients (Figure 4b).

### mRNA Expression for ENG, ALK1 and eNOS Genes in CACs

**Figure 5:**
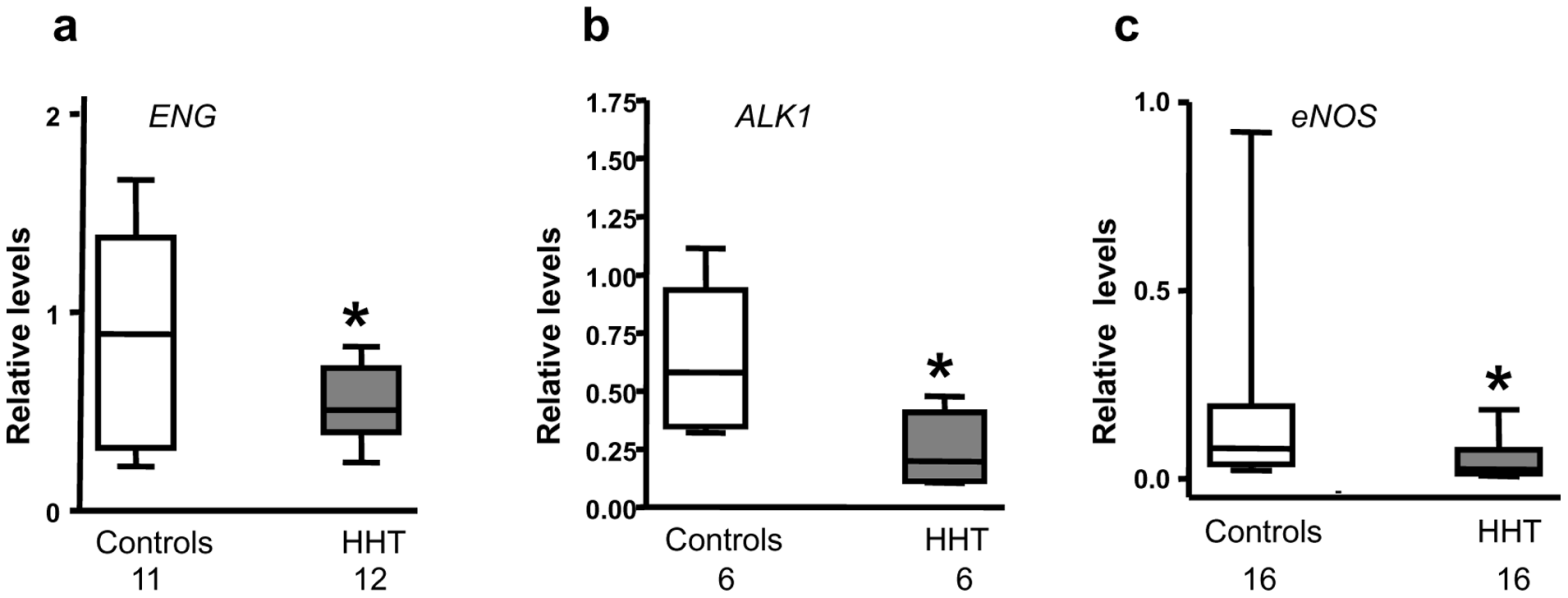
Decreased expression of *ENG, ALK1* and *eNOS* mRNAs in CACs from HHT patients. Relative mRNA levels for *ENG, ALK1* and *eNOS* genes in CACs were measured by real time RT-PCR with β-actin gene serving as an internal control. As shown in this figure, the mRNA levels of all 3 genes detected were significantly lower in CACs from HHT patients than that in CACs from healthy subjects. *p<0.05, compared with healthy controls.

The relative mRNA levels of *ENG*, *ALK1* and *eNOS* were determined by real time RT-PCR with the beta-actin gene serving as an internal control. As *ENG* and *ALK1*mRNA levels were found reduced in CACs from both HHT1 and HHT2, the results for these 2 genes were combined (HHT1 and HHT2). As shown in Figure 5a, *ENG* mRNA levels in CACs from HHT patients (0.5474±0.1903) were significantly reduced as compared with healthy controls (0.9838±0.6401, *p*<0.05). Similarly, *ALK1* mRNA levels were decreased in CACs from patients with HHT (0.2389±0.1499 vs. 0.6205±0.2887, HHT vs. Control, p<0.05; Figure 5b). *eNOS* mRNA relative levels were 0.0471±0.0479 in CACs from patients with HHT, which was significantly lower than 0.1715±0.2287 measured in CACs from healthy controls (*p*<0.05, Figure 5c). *eNOS* expression has also been shown to be reduced in ECs from patients with HHT [33].

## Discussion

HHT is an autosomal dominant vascular disorder. Both animal studies and clinical data have established that mutations in *ENG* or *ALK1* are responsible for the formation of the abnormal vasculature seen in the majority of patients with HHT [4]–[6]. Abnormalities of endothelial cells due to *ENG* or *ALK1* mutations are associated, and compatible with the vascular lesions in HHT [16]. CACs represent an important endogenous mechanism for vascular repair and regeneration, however their involvement in the pathogenesis of HHT has not been addressed.

In the present study, assessments of various aspects of CAC function were performed, and the results from patients with HHT were compared with those from healthy controls. We measured the expression of CD34, CD133 and VEGFR2 in freshly isolated PBMNCs from patients and healthy subjects using flow cytometry. An increase in the percent of PBMNCs expressing CD34+ in HHT patients was observed, although there were no differences seen in the fraction of PBMNCs staining positive for CD133 or CD34/VEGFR2. The total CAC number after seven days of culture did not differ significantly between the patients and healthy volunteers (data not shown). We speculate that the increase of circulating CD34+ cells in patients is a result of increased mobilization in response to pathological stimuli.

ENG and ALK1 have been shown to be essential for the maintenance of endothelial cell morphology and structure. Fernandez et al. have shown that circulating blood outgrowth endothelial cells (BOECs) derived from patients with HHT appear larger and rounder compared to the characteristic endothelial cobblestone shape of BOECs from control individuals. Furthermore, BOECs from individuals with HHT showed disorganization of the F-actin cytoskeleton, thought to be responsible for increased EC fragility and consequent capillary loss [16]. Vessel injury in HHT patients occurs randomly as a result of inflammation, trauma and other adverse insults, and therefore rapid and efficient repair of the endothelium is critical. CACs contribute to vascular repair through transdifferentiation into ECs as well as through the production of angiogenic growth factors. We demonstrated in this study that CACs from HHT patients not only displayed a disparate morphology, but also had a reduced ability to differentiate to EC linage cells. In addition, mRNA levels of eNOS, an important angiogenic agent, were found to be significantly lower in CACs from HHT patients.

In response to systemic stimuli, CACs are released from bone marrow into the circulation, and home to the injury site to exert their regenerative and angiogenic effects [17], [18], [34]. Using a murine myocardial infarction model, Van Laake et al have shown that transplantation of PBMNCs from healthy human donors into *ENG*+/− mice was sufficient to restore vessel formation and to improve heart function, but PBMNCs from HHT patients were not [35]. They proposed that the differential behavior in the mouse myocardial infarction model may be a result of defective homing, transdifferentiation, proliferation, or secretion of angiogenic factors of PBMNCs from HHT patients, and suggested that further studies on CACs from HHT patients was necessary. Using an in vitro migration assay, we found that CACs from either HHT patients had a reduced migration towards VEGF and SDF1 as compared to CACs from healthy volunteers, suggesting CAC mobilization in HHT patients is compromised.

It’s known that various cardiovascular disease states attenuate CAC number, function, and survival [30], [31], [36], [37]. However, little is known about the survival ability of CACs from HHT patients. We detected a higher level of basal apoptosis in cultured CACs isolated from HHT patients, although there was no difference in cell apoptosis after exposure to serum starvation or TNF α treatment between the patient and healthy groups. It is likely that 24-hour serum starvation and TNF α treatment are such powerful stimuli prompting cell apoptosis that the difference between the 2 groups is masked. Under conditions of serum starvation and exposure to TNF α, CACs from both healthy volunteers and patients with HHT showed greater rates of apoptosis (data not shown).

In this study, similar results were obtained in all experiments performed for CACs derived from HHT1 and HHT2 patients. It has been shown that ENG protein levels are decreased in ECs from both HHT1 and HHT2 patients, leading to aberrant TGF-β signaling, which is thought to be responsible for the similar cell behaviors observed in HHT1 and HHT2 ECs [16]. Although we did not explore the mechanism by which TGF-β signaling impacts CAC function, we found *ENG* mRNA was not only downregulated in HHT1 CACs but also in HHT2 CACs, similar to the finding in ECs. In conclusion, CACs from either HHT1 or HHT2 patients exhibited a reduced ability to differentiate to EC linage cells, decreased expression of eNOS, increased basal cell apoptosis and decreased migration towards VEGF and SDF1. These findings indicate that *ENG* or *ACVRL1* mutations not only impact the somatic endothelium, as previously reported, but may also result in CAC dysfunction. This may result in a reduced regenerative capacity of CACs to repair the vascular lesions seen in HHT patients.
